# Medical and Physical Intelligence

**Published:** 1821-11

**Authors:** 


### Medical and Physical Intelligence.

ROYAL *Society of Medicine at Marseilles.*—A PRIZE question, for a golden medal, has been proposed for the year 1822, by the above Society, on the two following subjects:—1. To determine the structure and functions of the spinal marrow. 2. To describe the nature, causes, symptoms, and treatment, of the diseases by which the spinal marrow is affected. The Society desires that clinical observation and pathological anatomy should be made the principal objects of the memoirs on these questions. They may be written either in Latin or French; and must be addressed, before the 1st of July, 1822, to the Secretary of the Society.

*Medical Funeral.*—THE celebrated CORVISART, well known by his writings to the English faculty, recently died. The following account of his funeral, which we extract from the *Journal des Debats* of the 22d September, may interest our medical readers:—

"The obsequies of M. le Baron de Corvisart, physician, took place to-day. In pursuance of the directions contained in his Will, his body was not taken to the church of St. Elizabeth, his parish, but was conveyed to his estate at Atys, where he desired that the religious ceremonies should be performed. A deputation from the Faculty of Medicine, in their doctor's robes, *(en habit doctoral,)* and nearly all the physicians of Paris, repaired this morning to his late residence, Rue de Vendome, for the purpose of attending his funeral. Mr. LEROUX, *doyen* of the physicians of Paris, pronounced a discourse over his coffin. The body, covered with the mantle of a doctor, and with the different orders which had been conferred upon the deceased, was afterwards placed in a hearse, to be taken to its destination, whither the deputation of the Faculty, and a great number of other persons, accompanied it."

*Origin of Vegetables.*—TURNIPS and carrots are thought indigenous roots of France; our cauliflowers came from Cyprus; our artichokes from Sicily; lettuce from Cos, a name corrupted into *Gause;* shallots, or eschallots, from Ascalon; the cherry and filbert are from Pontus; the citron from Media; the chestnut from Castana, in Asia Minor; the peach and the walnut from Persia; the plum from Syria; the pomegranate from Cyprus; the quince from Sidon; the olive and fig from Greece, as are the best apples and pears, though also found wild in France, and even here; the apricot is from Armenia.

*Benzoic Acid.*—Mr. ORGEL has ascertained that the petals of the *melilotus officinalis* contain a considerable quantity of benzoic acid, which might be extracted from them with equal facility and advantage. —*Tablettes Universelles, Mai* 1821.

*Nitrate of Potass.*—Mr. Tordeux, an apothecary at Cambrai, has had occasion to examine some hexaedral and circular crystals, which he found in a mass of the extract of cochlearia officinalis that had been lying-by for a considerable length of time. They consisted of nitrate of potash, which salt Mr. Tordeux has since been able to obtain from the same plant, by means of subacetate of lead, sulphuric acid, and concentrated alcohol. The presence of nitrate of potash in the cochlearia would explain the diuretic properties which that plant is known to possess.—*Memoire de la Societé de Cambrai.*

---

*Double Fetus.*—Twin children, closely connected at the chest, are said to be in the possession of Mr. James Barton, Dale End, Birmingham, and are in the highest state of preservation. There is only one set of organs to both children. They are of the usual size of children at nine months.—*Correspondent.*

---

*Singular Fetus.*—A male child was born, in May last, at the Hospital of la Maternité at Paris, with the whole surface of the body deeply wrinkled like that of a very old man. Its hands and feet are double the ordinary length, and are equally wrinkled. It has strong grey hair, and a beard of the same colour. In every other respect it enjoys perfect health, and is nursed in the hospital.—*Tablettes Universelles.*

---

*Medeography.*—Three vegetable productions, which might be advantageously employed in the practice of medicine, have been recently imported by Mr. Desmoges from Senegambia. 1. The *anadec,* a wood which the natives of Cape Vert Islands boil with rice, and employ with great success in cases of dysentery. 2. The *lémé lémé,* a violent purgative, the use of which is pretty general among the negroes of that part of the coast. It is burnt and reduced to powder, like coffee. A tea-spoonful of the powder, taken in a glass of water, is the ordinary dose. 3. The *bouganne,* the fruit of a tree which grows in Gazamarne and the neighbourhood of Joal. When boiled, it is looked upon as a specific remedy against the colic.—*Tablettes Universelles.*

---

*Accidental Poisoning by Oxymuriate of Lime.*—On Friday sennight, an infant child of Mr. Richards, of Chester, who had been left in charge of a servant-girl by its parents during the early part of the evening, having become very cross and uneasy, the girl took from the cupboard what she supposed to be a cordial occasionally given to the child, and administered a small portion of it. The infant presently became more violently affected, and remained in that state until the arrival of the mother, who, on being shown the bottle by the servant, immediately discovered the supposed cordial to be a poisonous mixture, called "bleaching liquid." Medical assistance was instantly summoned, but in vain. The infant continued to labour under excruciating agony; convulsions ensued, and about midnight it expired.

*Polyphagia.*—An American paper reports the death of a man, of whom several accounts have been recently published of his swallowing jack knives, bullets, marble, &c. On opening his body, twelve knives (all shut) were found in his stomach, one of them four inches and a half in length, and one a quarter wide; and among them the pocket-knife of the Philadelphia physician who had attended him. An authentic and detailed report of this very remarkable case will shortly appear in the American Medical Repository.

---

*Periodical Medical Publication in Spain.*—A Medical Journal, to be published every ten days, has been announced at Madrid, under the title of "*Las Decadas Medico Quirurgicas.* The objects of this periodical miscellany, are—1. To inform the profession and the public of all the discoveries and interesting facts relating to medicine and surgery in Spain and in foreign countries. 2. To give an impartial account of modern theories, medical doctrines, &c. 3. To convey intelligence respecting all endemic diseases. 4. An account of extraordinary cures. 5. Miscellaneous queries and observations, with an analysis of medical publications appearing in Spain, including the more important ones published among foreign nations.—On receiving the above announcement, we immediately wrote to our correspondent at Madrid to supply us with the work, without loss of time, for the use of the Medical and Physical Journal.

---

*Dr. Jackson and Dr. James Johnson.*—Dr. Johnson, of Spring Gardens, has addressed a letter to the Editor of one of the evening papers, on the subject of the yellow-fever now ravaging the southern coasts of Spain. We transcribe it without a comment.

THE PRESENT EPIDEMIA IN SPAIN.

*To the Editor of the Courier.*

*Spring Gardens; Oct. 8.*

Sir,—At a time when a most fatal pestilence is ravaging the southern coasts of Spain, and exciting such terror in the neighbouring states as to induce them to put in force the most rigid quarantine discipline, it may not be uninteresting to the British public to know that a countryman of their own, the veteran Dr. Jackson, now, I believe, on the verge of seventy, went voluntarily, and at his own expence, last year (1820), to the scene of this fever's devastations, in order to ascertain whether or not it was contagious, and what were the most effectual modes of treatment. He has just published a volume, containing very curious and important observations on this dreaded epidemic. It is a consolation to know that this very able and experienced physician has proved, very satisfactorily, that the disease is of a local origin; that it never was imported into those countries where it now rages; nor is it possible, almost, that it should ever be transported thence to any distance among the states in its vicinity. Popular prejudice having assigned the disease a foreign origin, popular terror has clothed it in properties of the most virulent contagion; and this last error has so divested the people of common humanity, that not only are the sick and wounded abandoned to their fate, but those who fly to the open country or mountains, (where, in fact, the malady cannot exist, much less propagate itself,) are treated like contaminated deserters from a pest-hospital, and either left to starve or put to death by their barbarous countrymen. It is consoling, however, to be certain (and it is most certain,) that a few weeks more,—that is, the approach of cold weather,—will cut off the sources of the epidemic, (which could not be the case where it a common contagion,) and thus annihilate, at least for a season, the disease itself.

In proof that the disease is not personally contagious, Dr. Jackson brings forward the following, among other authentic facts:—"In the year 1800, when upwards of ten thousand people died at Xeres de la Frontera, sixty persons were employed to bury the dead. The buriers entered the houses where the dead lay,—took the bodies in their arms, often, it is presumed, in a loathsome state,—put them into the carts in heaps, and drove them to the place of interment. None of the buriers were infected." A somewhat similar event happened in the year 1819, at Saint Lucar. The buriers of the dead having shamefully abused their office, the friars of the Franciscan convent most nobly volunteered the interment themselves. "The offer," says Dr. Jackson, "was accepted; and the friars, on entering on the duty they had thus imposed upon themselves, found the majority of the houses, or sick apartments, deserted, the bodies of the dead lying in various postures upon beds or on the floors. They wrapped them in sheets, or in such other covering as presented itself in the apartment, carried them to the bier in their arms, and afterwards in the bier on their shoulders to the grave. Not one of the meritorious band was attacked by the disease." In short, from the records of the past and the evidence of the present, there is not the least reason to doubt that the epidemic now ravaging Andalusia is of the same nature, though perhaps more violent in degree, as those which have, in autumnal seasons, appeared in the same places, at longer or shorter intervals, for centuries. The poison, or malaria, which produces the disease, is the product of the soil itself, elicited by particular seasons; and the fever so generated, if directly exported to other countries, could not there be propagated, for want of its primary pabulum.

Such is the opinion of Dr. Jackson, who visited the dying, and examined with his own hands the dead, so lately as in October 1820. It is also the opinion of the most enlightened English and French physicians who have visited the unfortunate theatre of the pestilence. Excepting the inconvenience, no harm apparently results from the quarantine precautions adopted by the neighbouring states; but the contagion conviction, impressed on the minds of those residing in and near the sickly towns and districts, gives rise to the most deplorable events, and deprives the unhappy sufferers of all succour or consolation from even their nearest friends or relatives! The operations of nature, however, and the revolution of the seasons, will soon put a period to the ravages of the epidemic.

James Johnson, M.D.

*Yellow-Fever in Spain.*—The details of the ravages committed by the contagious fever in Spain, are most lamentable. To guard against the propagation of this alarming malady, the French government has adopted the most rigorous precautions. A cordon of health is immediately to be extended along the frontiers of Spain, between which kingdom and France all communication is limited to one prescribed route, and travellers will be subjected to the performance of quarantine. All intercourse with the departments of l'Arreige and the Higher Pyrennees is rigorously interdicted for the present.

"A letter from Perpignan, dated the 18th instant, confirms the details already given relatively to the sad situation of Barcelona and a part of Catalonia. The number of persons who have quitted Barcelona is stated to be from thirty-five to forty thousand, who are scattered in different parts of that ill-fated province. Tortosa, and the whole shore of the Ebro, is a prey to this terrible scourge: Malaga, Cadiz, Xeres, and Port St. Mary, are not exempt from the infection. It is feared that the state of political affairs does not permit the government to deliberate with effect upon the necessary measures which such grievous circumstances require. Barcelona, it is said, is only occupied by individuals whom the hope of pillage has induced to remain. The lazaretto of that town is now empty: all those who were confined there have died. That of Barcelonetta is menaced with the same results.

"A letter from Mount Lewis, dated the 19th instant, announces that all Catalonia is in desolation, and that the streets of Barcelona are deserted; as the unhappy inhabitants have withdrawn to the mountains, from whence they are repulsed by the peasantry, and die of famine and misery. This letter confirms the previous accounts, that upwards of 67,000 passports were delivered before the establishment of the cordon of health. It was concealed from the inhabitants that this cordon would be formed at a very little distance from the town: as soon, however, as it was known to them, they committed sad excesses. The civil authorities had quitted the town.

A letter from Bayonne, dated the 22d, says:—"Advices have reached our authorities respecting the progress of the infection in Catalonia, which has determined them to adopt more rigorous precautions on the frontier of Navarre and Guipurzcoa. The customs' posts have been reinforced by three companies of troops of the line, which now form a strong cordon. The first detachment will be stationed at Hendaye, Biriatoco, the Pas-de-Behobia, and Olhetta; the second at Sare and St. Pée; the third at Ainhod and Itsazou.

"The Pas-de-Behobia is the only point by which communication with France will be permitted.

"These measures are at present merely precautionary, because but little communication exists between Catalonia and our neighbouring provinces. Even should contagious symptoms appear amongst us, the vigilance of the authorities and the fidelity of the troops would inspire us with security."

*State of Health at Malaga.*—We translate, from "El Universal," a Madrid paper, of the 25th September, the following extract respecting the present state of health of Malaga.

"*Malaga, 18th Sept.* The inhabitants of this town enjoy the most complete health. No contagious disorder seems to menace them at present; in proof of which fact we subjoin an account of the number of patients existing at present in the town, in the hospitals and lazarettos, all afflicted with common complaints, and of those who died yesterday.

"*Population.*—Patients known to exist yesterday, 60; died, 7.

"*Hospitals.*—Patients existing yesterday, 84; died, 1.

"*Lazarettos.*—0.

"*Harbour and Bay.*—One of the infected vessels, which had been ordered to Mahon, has returned; but will again be sent off immediately."

---

*Further Information respecting the Yellow-Fever.*—The conclusion of the official correspondence from Marseilles, inserted in another part of this Number, was communicated to us as the Journal was going to press. It appears that no fresh cases of fever have occurred since the last report,—thanks to the immediate and vigorous steps taken by the government to prevent the infection, even among the crews of ships in quarantine. The whole number of sick amounted to 23, of whom 12 died. The second *guard*, who had contracted the fever on-board the Danish vessel, and who had been landed with the corpse of his companion, as noticed in the above correspondence, fell also a victim to the disease in the lazaretto of Pomergue.

In the latest accounts received from Spain, the ravages committed by the epidemy are represented in the most formidable colours. Lerida, Barbastro, Tortosa, Mequinenza, and Trajôr, are all exposed to it, from want of proper measures of sequestration. Saragossa having also been threatened, the Board of Health have been under the necessity of resorting to rigorous precautions. Various extracts of letters of the 3d and 5th instant (October,) represent the epidemic disease as having increased in severity. "On the 23d and 24th of September, 260 persons died, exclusive of children. The whole population of Tortosa is said to have been swept away by the disorder. A cordon has been formed on the frontiers of France and Catalonia. The inhabitants of the latter province are flying from their homes. The situation of Barcelona becomes daily more distressing. It was hoped (observes the writer,) that the last days of September would, with a cooler temperature, bring the cheering prospect of freedom from our sufferings, and put an end to the devouring malady that afflicts us; but we are disappointed: the victims of the yellow-fever increase in number. Ninety died yesterday, and 600 new cases have been declared."

The northern powers have lately issued orders for subjecting every vessel coming from Spain to the most rigorous quarantine, and for rejecting all those on-board of which any case of fever may have occurred.

Drs. Pariset, Mazet, and Bailly, three of the physicians deputed by the French government to proceed to Spain, to inquire into the nature and progress of the epidemy, are said to have begun their labours.

*Paris*, 13*th October*.—Seven days ago, a Danish ship was stranded in the night, about two leagues from Marseilles. Three members of the Board of Health instantly repaired to the spot, when they learned that two of the crew had died of the fever on the passage. The ship was accordingly burned without delay, and the men conveyed to the lazaretto.

---

*Juridical Medicine.*—Two inquisitions have been taken, in the course of last month, at Mildenhall, before Mr. Wayman, the coroner for the liberty of Bury, in consequence of premature death occurring in an adult and an infant, the causes of which merit the attention of the medical profession. It appeared upon evidence, that the former, named John Harris, had eaten some honey from the honey-comb, and that a bee concealed in it getting into his throat, he died almost immediately from suffocation. The second, Mary Bacon, fell with her face upon a quantity of slacked lime, when a particle of it getting into the wind-pipe, produced inflammation of the lungs and sloughing of the trachea, of which this infant died. The verdict in each case was "Accidental Death."

---

*University of Edinburgh.*—The medical class for the ensuing session was opened as follows:—

Dietetics, Materica Medica, and Pharmacy, Wednesday, Oct. 31, at eight o'clock, Dr. Duncan, jun.

Practice of Physic, Wednesday, Oct. 31, at nine o'clock, Dr. Home.

Chemistry and Chemical Pharmacy, Wednesday, Oct. 31, at ten o'clock, Dr. Hope.

Theory of Physic, Wednesday, Oct. 31, at eleven o'clock, Dr. Duncan, sen. and Dr. Alison.

Anatomy and Pathology, Wednesday, Oct. 13, at one o'clock; and Principles and Practice of Surgery, Wednesday, Nov. 14, at four o'clock; Dr. Monro.

Theory and Practice of Midwifery, Thursday, Nov. 13, at three o'clock, Dr. Hamilton.

Clinical Medicine, Tuesday, Nov. 13, at four o'clock, Dr. Graham and Dr. Alison.

Clinical Surgery, Monday, Nov. 5, at five o'clock, Mr. Russell.

Practical Anatomy, under the superintendance of Dr. Monro.

During the summer season, Lectures will be given on the following branches of education:—Botany, by Dr. Graham; Midwifery, by Dr. Hamilton; Clinical Lectures on Medicine, by Dr. Graham; Clinical Lectures on Surgery, by Mr. Russell; and Universal History, by Sir W. Hamilton.

Attendance will be given in the Library every lawful day, from ten till three o'clock, to enrol the names of the students in the Album, which is the only legal record of their attendance in the University.

---

*Cæsarean Operation.*—At the meeting of the Royal Academy of Medicine at Paris of the 7th of July last, Mr. Beclard, one of the most distinguished surgeons of that capital, reported that he had, the day before, performed the Cæsarean operation, and that both the mother and child were doing well. The incision was made in the direction of the linea alba. The report continues favourable down to the 10th of July, after which day we have received no further account.

---

*A Reply to Dr. James Johnson's Letter, inserted in the present Number.*—We hold it to be part of our duty, as journalists, to avail ourselves of every transient incident that can benefit the health of mankind, in every part of the globe where our periodical labours circulate: we therefore cannot refrain from making use of a testimony, which, though not from a professional person, yet, as mere matter of fact, is entitled to as much, or perhaps more, attention than the like attestations of professional gentlemen, whose minds are too often warped by preconceived opinions. An article has appeared in the Liverpool and London newspapers, signed by a private gentleman, of the name of Chevalier, stating, in reply to Dr. James Johnson's letter regarding the non-infectious nature of yellow-fever, that he can affirm, of his own knowledge, that the fever in question was introduced into Cadiz, in the year 1800, by a ship from Philadelphia, on-board of which some persons had died of it during the voyage. This vessel was at first put into quarantine, but was soon after released through the influence of a merchant with a magistrate, both of whose names he gives; and, from the landing of the people, and their baggage, belonging to this ship, the infection was traced on that occasion.

We are assured, on the authority of Señor Larrea, the Spanish consul residing in this country, part of whose family resided at Malaga, that this dreadful epidemic was introduced there in 1803, by a vessel from St. Domingo, which, by the interference of interested people, was excused from quarantine, though it was well known that the disease was on-board at the time. From Malaga, the fever spread on the following year, 1804, to Gibraltar, where 6000 inhabitants fell victims to it.

---

The unexpected demise of Don Fernando Millares, the governor of Malaga, which took place on the 27th of September, has created the liveliest alarm among the inhabitants of that town; two thousand of whom immediately left it, under an apprehension that the complaint of which that officer had died was the yellow-fever.—*Madrid Gazette, October 2d.*

*Preservative against the Yellow-Fever.*—A PHILANTHROPIC person has addressed a letter, written in sober earnestness, to the superior political chief of the province of Arragon, on the subject of the yellow-fever which devastates part of that province, recommending the use of a mixture of thirty-five drops of nitric acid and twelve ounces of spring water, as an infallible preservative against that formidable malady. The quantity of the diluted acid to be taken for that purpose, is two ounces three times a-day.—*El Universel, October 3d.*

---

*Spanish Apothecaries.*—A SINGULAR retrospective decree of the Faculty of Pharmacy in Madrid, has lately been issued, by which every person practising pharmacy or keeping a chemist's shop, who has not reached the age of twenty-five years, is enjoined to attend courses of lectures at the Royal College up to that age, with a view to his being re-examined previously to obtaining a fresh licence to practise.

---

" *Ecole Physico-Pathologique.*"—A-PROPOS of Dr. BROUSSAIS. Our readers will not be displeased to see in what estimation the theory and practice of this professor are held by some of his brethren in Paris. We copy the following *jeu-d'esprit* from a highly respectable Journal of July last. Those who have read the very able exposition of Dr. Broussais' system in Dr. James Johnson's Review, and our own feeble endeavours to set a real value on the doctrine of this foreign professor in the Medical and Physical Journal of last month, will easily comprehend the drift of the following translation in reference to one who contends that all complaints arise from the stomach, and that lemonade and leeches are to cure every complaint.

" A distinguished man of letters, who is publishing a new edition of MOLIERE, aware of the ridiculous appearance of some modern physicians, has determined to make some variations in the celebrated scene of the comedy of that author, entitled " Le Malade Imaginaire." The following is a specimen:—

*Toinette (dressed as a physician.)* Let me feel your pulse.—Come, come, beat as you ought. Ah! I'll soon make you go right. Faith! this pulse is mightily impertinent. I see plainly it does not know whom it has to deal with. Who is your attending physician, sir?

*Argan.* Monsieur Purgon.

*Toin.* That name is not to be found in my pocket-book, amongst the physicians of eminence. What did he say was the matter with you?

*Arg.* He says my liver is affected: others contend that it is the spleen.

*Toin.* Pooh! pooh! They are a pack of *ignoramus*. It is your stomach, sir, that is affected.

*Arg.* The stomach?

*Toin.* Yes! the stomach. What do you feel?

*Arg.* I have occasionally the head-ach.

*Toin.* Exactly so. The stomach.

*Arg.* I sometimes see double;—I have a veil over my eyes.

*Toin.* The stomach!

*Arg.* And a great difficulty in breathing.

*Toin.* The stomach again!

*Arg.* I suffer from great langour and lassitude in all my limbs.

*Toin.* The stomach!

*Arg.* And I now and then feel pain in my feet, in my heels, and toes, as if I had the gout.

*Toin.* Exactly so. It is the stomach! You eat with good appetite?

*Arg.* Yes, sir.

*Toin.* The stomach! You like to drink a small quantity of wine.

*Arg.* Yes, sir.

*Toin.* The stomach! You feel rather drowsy after dinner, and delight in taking a nap?

*Arg.* Oh yes, sir.

*Toin.* The stomach—the stomach, I tell you! What has your physician desired you to eat?

*Arg.* He desired me to take some soup.

*Toin.* *Ontologist*!*

*Arg.* And chicken.

*Toin.* Fatalist!

*Arg.* And occasionally veal.

*Toin.* Incendiary!

*Arg.* Broth.

*Toin.* Murderer!

*Arg.* Some fresh eggs.

*Toin.* What an assassin!

*Arg.* And stewed prunes in the evening.

*Toin.* Incendiary, and murderous diet of the ontologistic school!

*Arg.* And, above all, he desired me to put water to my wine.

*Toin.* *Ignorantus, ignoranta, ignorantum!* This is not the way of a *medecin physiologiste.* You must drink pure water, which is indeed too nourishing as it is: it will calm the gastro-intestinal irritation under which you labour; and thin your blood, which is now too dense. You'll apply five hundred leeches to the pit of the stomach, which will remove the *gastro-enteritis* in the twinkling of an eye. Don't you see that "irritation is preying heavily on the mucous membrane of your stomach, from whence it extends its malignant power over all the organs of your economy, through sympathetic irradiations?"† Be assured that your physician is an *ontologist,* who will believe only what he sees. I will come again to see you in a fortnight, if you are alive; and, above all, rest assured that, if I have abused

* An appellation of contempt adopted by Dr. Broussais, to designate all those physicians who seem to have identified or personified the different causes producing diseases,—such as the gouty principle, the scrofulous humour, &c.

† This is not a parody, but the real language, of Dr. Broussais, repeatedly met with in his work on Phlegmasiæ, and the "Examen des Doctrines Medicales," which we reviewed in our last Number.

my brethren of the profession, it was not through envy, but for the good of mankind. How superior to them all the *doctrine physiologique* makes me!—a doctrine which is destined to do more good even than the immortal discovery of Jenner.*

* This is verbatim the manifesto of Dr. Broussais. " Ma doctrine doit avoir prochainement sur la population une influence plus marquée que la découverte de la vaccine." *Risum teneatis Pisoni !*

### METEOROLOGICAL JOURNAL.

By Messrs. William Harris and Co. 50, Holborn, London.

*From September* 20 *to October* 19, *inclusive.*

| Day of Month. | Moon. | Rain gauge | THERM. | | | BAROM. | | DeLuc's Hygro. | | WIND. | | ATMOSPHERIC VARIATION. | | |
|---|---|---|---|---|---|---|---|---|---|---|---|---|---|---|
| Sep. | | | | | | | | | | | | | | |
| 20 | | | 56 | 62 | 55 | 29.95 | 29.82 | 62 | 60 | W | W | Cloudy | Rain | Cloud. |
| 21 | | .42 | 58 | 63 | 58 | 29.78 | 29.76 | 61 | 62 | SW | SW | Rain | Fine | Rain |
| 22 | | | 62 | 67 | 59 | 29.79 | 29.78 | 58 | 59 | E | ESE | Cloudy | | Foggy |
| 23 | | | 62 | 65 | 54 | 29.67 | 29.67 | 58 | 59 | ESE | ESE | Foggy | Cloud. | Fine |
| 24 | | .11 | 56 | 64 | 51 | 29.64 | 29.91 | 54 | 55 | NW | NNW | Fine | Sho'ry | Fine |
| 25 | | | 54 | 65 | 54 | 30.01 | 30.02 | 53 | 61 | NW | WSW | Cloudy | Fine | Cloud. |
| 26 | ● | | 59 | 64 | 59 | 30.00 | 30.00 | 66 | 65 | W | SW | Cloudy | Fine | Cloud. |
| 27 | | .13 | 60 | 66 | 59 | 29.88 | 30.00 | 60 | 59 | NW | W | Rain | Fine | Cloud. |
| 28 | | .19 | 61 | 68 | 58 | 29.71 | 29.53 | 55 | 51 | W | W | Cloudy | Fine | Rain |
| 29 | | | 58 | 60 | 46 | 29.65 | 29.71 | 51 | 49 | W | NW | Fine | | |
| 30 | | | 50 | 58 | 53 | 29.85 | 29.92 | 47 | 46 | W | W | Fine | | |
| Oct. | | | | | | | | | | | | | | |
| 1 | | | 57 | 63 | 49 | 29.68 | 29.98 | 57 | 49 | W var. | NW | Fine | | |
| 2 | | | 53 | 62 | 50 | 30.15 | 30.12 | 47 | 48 | NW | W | Fine | | Foggy |
| 3 | | .15 | 54 | 65 | 52 | 29.97 | 29.80 | 53 | 63 | SW | WSW | Rain | Sho'ry | Cloud. |
| 4 | ☾ | .34 | 58 | 65 | 44 | 29.64 | 29.48 | 64 | 59 | SW | SW | Rain | | |
| 5 | | | 48 | 61 | 49 | 29.85 | 29.91 | 51 | 60 | NW | NW | Fine | | |
| 6 | | | 54 | 60 | 55 | 30.00 | 30.08 | 60 | 59 | NW | SW | Fine | | |
| 7 | | .09 | 60 | 62 | 48 | 30.09 | 30.03 | 61 | 59 | WSW | SW | Cloudy | Fine | |
| 8 | | .21 | 53 | 63 | 49 | 30.09 | 30.21 | 55 | 51 | WNW | NNW | Rain | Fine | |
| 9 | | | 55 | 64 | 47 | 30.17 | 30.21 | 53 | 50 | SW | SSE | Foggy | Fine | |
| 10 | | | 50 | 61 | 48 | 30.13 | 30.91 | 50 | 53 | SE | ESE | Fine | | |
| 11 | ○ | .10 | 54 | 64 | 54 | 29.77 | 29.65 | 55 | 61 | SE | SSE | Fine | | Sho'ry |
| 12 | | | 57 | 65 | 50 | 29.82 | 30.13 | 55 | 55 | NNW | NW | Fine | | |
| 13 | | | 52 | 57 | 47 | 30.27 | 30.36 | 57 | 53 | NE | NE | Foggy | Fine | Foggy |
| 14 | | | 50 | 55 | 44 | 30.26 | 30.28 | 51 | 53 | SW | WSW | Foggy | Fine | |
| 15 | | .17 | 48 | 52 | 44 | 30.18 | 30.21 | 51 | 55 | NE | NNE | Rain | Fine | |
| 16 | | | 46 | 54 | 45 | 30.16 | 30.21 | 53 | 55 | NE | NNE | Fine | | |
| 17 | | | 48 | 54 | 44 | 30.17 | 30.10 | 59 | 60 | WNW | W | Fine | | Foggy |
| 18 | ☽ | .21 | 48 | 52 | 45 | 29.99 | 29.95 | 59 | 57 | WNW | N | Rain | Cloud. | |
| 19 | | | 48 | 55 | 45 | 29.81 | 29.67 | 60 | 58 | W | SW | Fine | | Cloud. |

The quantity of rain fallen in the month of September, was 2 inches and 15-100ths.

